# An Internet-Based Intervention for Depression in Primary Care in Spain: A Randomized Controlled Trial

**DOI:** 10.2196/jmir.5695

**Published:** 2016-08-26

**Authors:** Jesús Montero-Marín, Ricardo Araya, María C Pérez-Yus, Fermín Mayoral, Margalida Gili, Cristina Botella, Rosa Baños, Adoración Castro, Pablo Romero-Sanchiz, Yolanda López-Del-Hoyo, Raquel Nogueira-Arjona, Margarita Vives, Antoni Riera, Javier García-Campayo

**Affiliations:** ^1^ Faculty of Health Sciences and Sports University of Zaragoza Zaragoza Spain; ^2^ Primary Care Prevention and Health Promotion Research Network, RedIAPP, ISCIII Madrid Spain; ^3^ Aragon Institute for Health Research (IIS Aragon) Zaragoza Spain; ^4^ London School of Hygiene and Tropical Medicine London United Kingdom; ^5^ Aragon Health Sciences Institute Zaragoza Spain; ^6^ Mental Health Clinical Management Unit, Institute of Biomedical Research of Malaga (IBIMA), Regional Universitary Hospital Carlos Haya, University of Malaga Málaga Spain; ^7^ University of Balearic Islands Palma de Mallorca Spain; ^8^ Universitat Jaume I Castellón de la Plana Spain; ^9^ Ciber Fisiopatología Obesidad y Nutrición (CB06/03) Instituto Salud Carlos III Madrid Spain; ^10^ University of Valencia Valencia Spain; ^11^ University of Zaragoza Zaragoza Spain; ^12^ University of Malaga Málaga Spain; ^13^ University Hospital Miguel Servet Zaragoza Spain

## Abstract

**Background:**

Depression is the most prevalent cause of illness-induced disability worldwide. Face-to-face psychotherapeutic interventions for depression can be challenging, so there is a need for other alternatives that allow these interventions to be offered. One feasible alternative is Internet-based psychological interventions. This is the first randomized controlled trial (RCT) on the effectiveness of an Internet-based intervention on depression in primary health care in Spain.

**Objective:**

Our aim was to compare the effectiveness of a low-intensity therapist-guided (LITG) Internet-based program and a completely self-guided (CSG) Internet-based program with improved treatment as usual (iTAU) care for depression.

**Methods:**

Multicenter, three-arm, parallel, RCT design, carried out between November 2012 and January 2014, with a follow-up of 15 months. In total, 296 adults from primary care settings in four Spanish regions, with mild or moderate major depression, were randomized to LITG (n=96), CSG (n=98), or iTAU (n=102). Research completers at follow-up were 63.5%. The intervention was Smiling is Fun, an Internet program based on cognitive behavioral therapy. All patients received iTAU by their general practitioners. Moreover, LITG received Smiling is Fun and the possibility of psychotherapeutic support on request by email, whereas CSG received only Smiling is Fun. The main outcome was the Beck Depression Inventory-II at 3 months from baseline. Mixed-effects multilevel analysis for repeated measures were undertaken.

**Results:**

There was no benefit for either CSG [(B coefficient=-1.15; *P*=.444)] or LITG [(B=-0.71; *P*=.634)] compared to iTAU, at 3 months. There were differences at 6 months [iTAU vs CSG (B=-4.22; *P*=.007); iTAU vs LITG (B=-4.34; *P*=.005)] and 15 months [iTAU vs CSG (B=-5.10; *P*=.001); iTAU vs LITG (B=-4.62; *P*=.002)]. There were no differences between CSG and LITG at any time. Adjusted and intention-to-treat models confirmed these findings.

**Conclusions:**

An Internet-based intervention for depression combined with iTAU conferred a benefit over iTAU alone in the Spanish primary health care system.

**Trial Registration:**

Clinicaltrials.gov NCT01611818; https://register.clinicaltrials.gov/prs/app/action/SelectProtocol? selectaction=Edit&uid=U0001NPQ&ts=2&cx=gctdh2&sid=S0003KJ6 (Archived by WebCite at http://www.webcitation.org/6jbsUvUDz)

## Introduction

Depression is the most prevalent cause of illness-induced disability worldwide [1]. It is among the most common reasons for consulting a general practitioner (GP), and it carries considerable personal and economic burden [2]. Antidepressants are a common form of treatment for depressive patients in primary care (PC) [3], but many patients would also like to receive psychotherapy [4]. Psychological treatments for depression are shown to be effective in PC, especially when GPs refer patients for treatment [5]. There is evidence that psychological treatments achieve results as effective as those achieved by antidepressant medication, reducing the number of physician consultations and hospital days, and obtaining better results in adherence, relapse prevention, and reducing chronicity [6].

Nevertheless, delivering face-to-face psychotherapeutic interventions to a population is challenging given the lack of specialized resources [7], so there is a need for other alternatives that allow these interventions to be offered. One feasible alternative is Internet-based psychological interventions [8]. The Internet offers a way of providing psychological treatments for depression [9] that may even attract people who are reluctant to use traditional mental health services [10] because of barriers such as possible stigmatization processes [11]. In general, Internet-based psychological treatments seem to be effective for the treatment of depression. Although the effects seem to be more favorable for guided or assisted interventions [12-14], stand-alone Internet-based treatments for depression have also shown to be effective [15].

Until now, there have been no studies on the effectiveness of Internet-based treatments for depression in the context of PC in Spain. Therefore, the main objective of this study was to compare the effectiveness of a low-intensity therapist-guided (LITG) Internet-based program and a completely self-guided (CSG) Internet-based program with improved treatment as usual (iTAU) care for the treatment of major depression in PC in Spain.

## Methods

### Hypotheses

The main hypothesis was that both Internet-based interventions, CSG and LITG, would be more effective in reducing depressive symptoms than iTAU, in the context of PC at 3 months after baseline. A secondary hypothesis was that, in the context of PC, where there is a more frequent and closer contact with the GPs, the offer of additional help is unlikely to improve outcomes when using Internet-based interventions.

### Design

This study was a multicenter, three-arm, parallel, randomized controlled trial (RCT). Adults presenting with depressive symptoms in PC were randomized to receive either iTAU from their GP or an Internet-based intervention program (Smiling is Fun) for depression, in this case, either with psychotherapist support (LITG) or without it (CSG). The trial protocol of the study [16], the manual used to implement the program [17], and a study on expectations of depressed PC patients have already been published [18,19].

### Recruitment of Participants and Baseline Assessment

We recruited patients with major depression, aged 18-65 years, able to understand and read Spanish, with mild or moderate severity symptoms according to the Spanish Beck Depression Inventory-II (BDI-II) (14-19: mild depression; 20-28: moderate depression) [20], with symptoms lasting longer than 2 weeks, with access to Internet at home, and having an email account. Major depression was identified using the MINI International Neuropsychiatric Interview 5.0, which can establish major depression diagnoses according to the Diagnostic and Statistical Manual, version IV (DSM-IV) and International Classification of Diseases [21,22]. We excluded patients who had been receiving any psychological treatment during the previous year, those with severe psychiatric disorder in Axis I (eg, alcohol/substances abuse or dependence, psychotic disorders, dementia), and patients with severe depression (score ≥29 on the BDI-II), who were referred by their GPs for treatment.

Participants were recruited in PC settings, between November 2012 and January 2014, in the Spanish regions of Aragon, Andalusia, the Balearic Islands, and Valencia. GPs identified potential participants through using a case-finding questionnaire. Eligible individuals were then interviewed in the clinic within the following 3 days by an independent researcher, who assessed inclusion and exclusion criteria, using the MINI psychiatric interview and other questionnaires. Informed consent to enter the trial was sought from patients who fulfilled study criteria, followed by randomization, carried out by an independent researcher. Patient safety was systematically monitored. The Ethical Review Board of the regional health authority approved the study on April 7, 2010 (ref: PI10/01083).

### Randomization, Concealment, and Blinding

Participants were individually randomized using blocked randomization to one of the three groups. Blocks were administered in each of the regions, using a computer-generated random number sequence. A person who had no other involvement in the study managed the random allocation to groups. This procedure was implemented through a remote central telephone line. The sequence was concealed until all individuals had been randomized. Although patients were not informed of the group allocation, the nature of the intervention meant that it was virtually impossible to keep this completely blind. Study personnel conducting the outcome assessments were blind to the participants’ allocation.

### Follow-Up

Collection of follow-up data took place between March 2013 and June 2015. Participants were assessed online at 3 (time-1), 6 (time-2), and 15 (time-3) months post-baseline assessment. These moments deviated from the registered protocol. Post-treatment evaluation was stated at 3 months after the beginning of the intervention in order not to favor participants in the intervention conditions who could take longer than the estimated intervention duration comparing to control group. By setting the same evaluation moments for all participants, we ensured that measurements would be comparable. Participants were sent an email with a link to an online platform that hosted the questionnaires. No other protocols were used to increase compliance with the research data collection, but a phone call was made before each wave assessment to increase response rates.

### Control Group

All the patients included in the study (whether in the control or intervention arms) received iTAU. This treatment was provided by their GPs, who had previously received a 3-hour training program to update their knowledge on how to diagnose and treat depression in primary care, based on the National Institute for Health and Care (NICE) guidelines [23]. The training mostly dealt with the appropriate use of antidepressants. In case of suicide risk or severe social dysfunction, or if worsening of symptoms was detected, patients were referred to mental health facilities.

### Intervention Groups

Smiling is Fun is an Internet-delivered, self-help program for the treatment of depression, based on similar programs that have proven effective in other countries [24]. The program consists of 10 cognitive behavioral therapy modules, covering different psychological techniques for coping with depression. These modules need to be completed in a sequential way. The program recommends working on every module for at least a week, with the following modules: (1) Medication management (psychoeducation I), (2) Sleep hygiene (psychoeducation II), (3) Motivation for change (motivation), (4) Understanding emotional problems (psychoeducation III), (5) Learning to move on (behavioural activation), (6) Learning to be flexible (cognitive therapy), (7) Learning to enjoy (positive psychology I), (8) Learning to live (positive psychology II), (9) Living and learning (positive psychology III), and (10) From now on, what else? (relapse prevention). A more specific and detailed description of the module contents can be found elsewhere [16,17].

Patients in the intervention groups were allocated to LITG or to CSG Internet-based programs. In LITG, 4 trained psychotherapists randomly contacted the patients by email to offer help with any difficulties or problems encountered when using the program. Patients could ask the psychotherapists questions or advice via email messages with a maximum of three contacts over the treatment period. They could also ask a technician for help to resolve problems of a technical nature. In CSG, there was no contact with any therapist, and only technical questions could be asked regarding the computer program.

To maximize adherence, if participants did not access the program for a week, they received an automated email encouraging them to use the program and to complete the tasks for each module. In addition, the program offered continuous feedback to the users on their progress via (1) a self-monitored activity report, providing feedback on how their mood was related to the activities performed, (2) the calendar, providing feedback about homework and tasks already completed, and (3) graphs and other feedback about activity levels, emotional distress, and negative and positive emotionality.

Among those patients on medication, GPs and patients were advised not to increase dosages in any of the three groups (iTAU, CSG, LITG), but decreasing medication was permitted.

### Instruments

#### Demographic Variables

We gathered sociodemographic data such as age, sex, living with family or alone, level of studies (university vs secondary or less), employment (employed vs unemployed), and income according to national minimum wage, as well as clinical variables such as taking antidepressant medication (yes vs no) and the number of GP visits in the previous 12 months.

#### Outcomes

The Spanish version of BDI-II [19], as a continuous variable, was used as the primary outcome measure at time 1 (3 months after baseline). The BDI-II is one of the most widely used instruments to evaluate presence and severity of depressive symptoms. It is a self-reported measure, which includes the cardinal cognitive, emotional, and somatic symptoms of depression, and it can be linked to diagnosis from the DSM-IV. The studies published show good agreement between BDI-II and the clinical diagnosis of depression, and good psychometric properties for the scale [25,26]. Scores can range from 0-63.

Secondary outcomes included the visual analogue scale (VAS) of the EuroQol (EQ-5D) [27], in its Spanish version [28], and the Short Form Health Survey (SF-12v1) [29], in its Spanish version [30], as measures of health-related quality of life and functioning. The VAS is a vertical line on which the best and worst possible health states are scored 100 or zero respectively. The SF-12 scoring algorithm yields a physical component scale and a mental component scale, and both were used as continuous variables applying Spanish norms, with a mean of 50 (SD 10) [30].

### Sample Size

Estimated sample size in protocol was 450 participants [16], but there were recruitment problems in one of the four participant regions, Valencia, where there were multiple stakeholders, so the recruitment was delayed for more than one year. In view of this, the steering committee of the project decided to rule out recruitment in that region, repeating the process of sample size calculation, according to the new situation. This new power calculation was based on testing differences between LITG Internet-based program and iTAU care alone, and we based our sample size estimation on an expected difference in the primary outcome of at least 0.5 standard deviation (SD) [14]. This size has been considered as a clinically relevant criterion [24,31]. Previous PC studies of depressed patients in England and Spain have found BDI-II means and SDs of 22 and 12, respectively, but as our inclusion cut-off points attenuate variability, we used an SD of 6. Thus, a difference of 3 points across these two groups was our target (around 15%). In order to detect this difference between LITG and iTAU, assuming a common SD of 6 points, a 5% significance level and a statistical power of 80%, we needed 63 subjects in each group. We expected a dropout rate of around 30% [12,32], so we inflated the numbers to reach a total sample size of around 300 patients (100 per arm). This change in the number of participants was more realistic for the new situation of recruitment.

### Data Analysis

First, descriptive data were compared to assess the balance of a number of variables across arms at baseline. All analyses followed a pre-specified plan [16], based on the Consolidated Standards of Reporting Trial (CONSORT) guidelines [33] (Multimedia Appendix 1). The primary between-group analysis was carried out on an intention-to-treat basis for BDI-II total scores, using a multilevel mixed-effects analysis for repeated measures, and calculating regression coefficients (B), unadjusted and adjusted for baseline scores, sex, and age [34]. Sensitivity analyses were conducted to assess the effects of missing data. Missing values were replaced by multiple imputations based on chained equations, after ensuring that data were missing at random [35]. Secondary analysis comprised comparisons of SF-12 Mental and Physical subscale scores, as well as EuroQol VAS scores using the same analytical strategy. Effect sizes between groups were calculated by means of Hedge’s *g*, and group by time interactions (3 groups and 4 time points) through chi-square tests, unadjusted and adjusted for baseline, sex, and age, with the associated degrees of freedom of (r-1) x (c-1), where r is the number of groups and c is the number of time points. We also performed a Complier Average Causal Effect (CACE) analysis to assess the impact of the number of sessions on the outcome. We theoretically defined compliance as attendance at >6 sessions, but we also performed a parallel assessment of the impact for each session added separately.

We used two-sided tests at the 5% significance level, taking into account Bonferroni’s criterion whenever there were multiple comparisons. All the analyses were performed with Stata 12.

## Results

**Figure 1:**
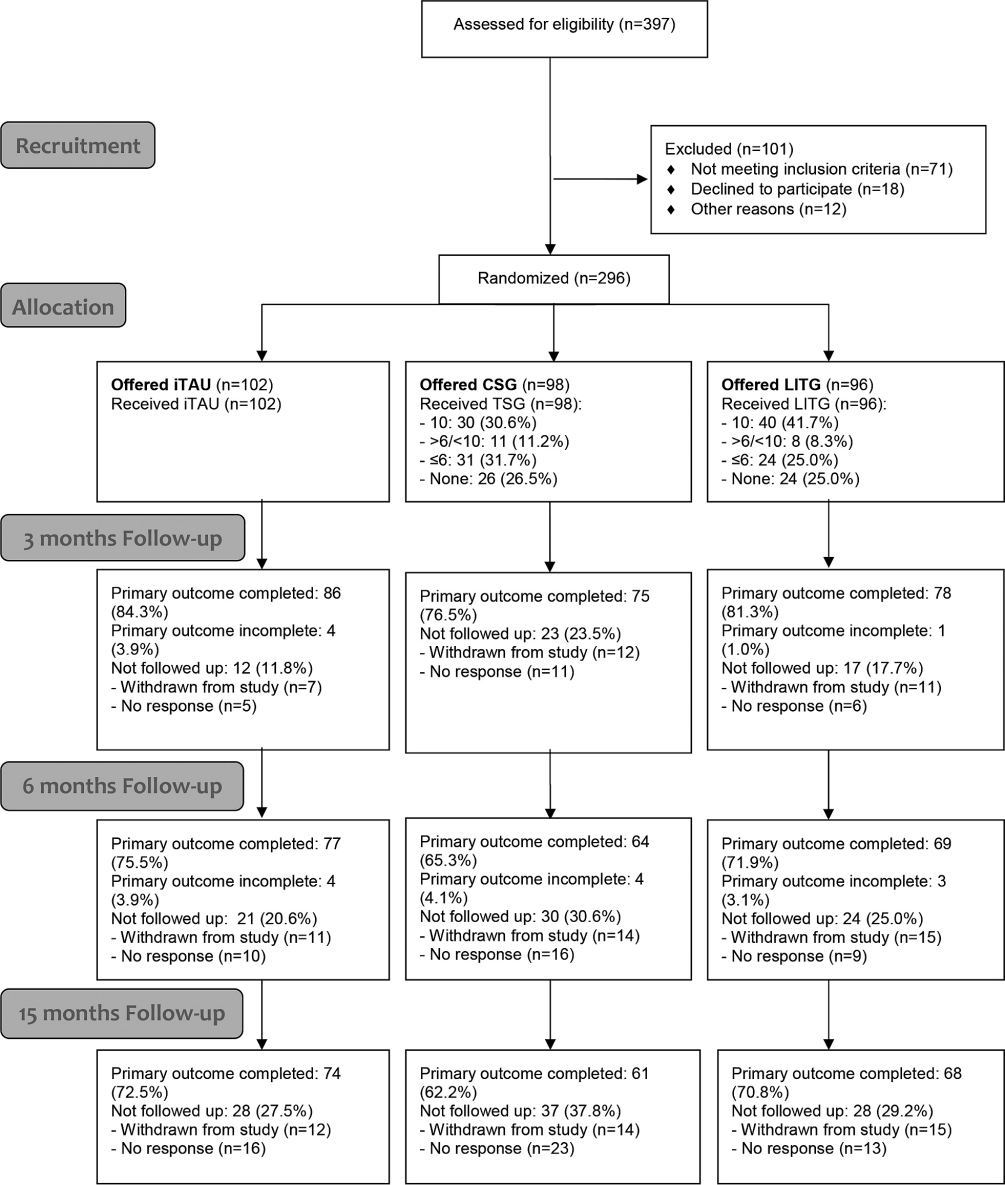
Flow diagram.

A total of 46 GPs took part in the study. Of 397 potential participants, 296 were randomized (see Figure 1), with 102 allocated to iTAU, 98 to the CSG, and 96 to the LITG. The randomized groups were well balanced in all variables at baseline (Table 1). Recruitment varied across regions: 126 participants from Aragon, 106 from Andalusia, 44 from the Balearic Islands, and 20 from Valencia. Follow-up primary outcome data were obtained for 239 (80.7%) of the participants at Time 1, 210 (70.9%) at Time 2, and 203 (68.6) at Time 3. There were no significant differences among groups in terms of attrition rate (iTAU=34.3%; CSG=41.8%; LITG=33.3%; χ^2^_2_=0.42; *P*=.812). Only age was significantly related to attrition (at Time 3) [completers (n=203): mean 44.28 years (SD 10.22) vs missing (n=93): mean 39.99 years (SD 10.01); *P*=.001]. No baseline-level differences in other sociodemographic or primary or secondary outcomes were observed between completers and non-completers at different waves, so dropouts were considered as to be random [36]. The median depression severity across all groups in BDI-II at baseline was 23, which broadly equated with a depression of moderate severity [20].

**Table 1:** Baseline characteristics of participants across groups.

Characteristics at baseline			iTAU, n=102	CSG, n=98	LITG, n=96
**Sociodemographics**					
	Age, mean (SD)		43.04 (9.66)	42.57 (11.94)	43.19 (9.30)
	Sex female, n (%)		76 (74.5)	72 (73.5)	76 (79.2)
	Living with family, n (%)		92 (90.2)	90 (91.8)	82 (85.4)
	University education, n (%)		30 (29.4)	29 (29.6)	32 (33.3)
	Employed, n (%)		54 (52.9)	51 (52.0)	52 (54.2)
	**Income,** n (%)				
		<1 national minimum wage	27 (26.5)	34 (34.7)	22 (22.9)
		1-2 national minimum wage	42 (41.2)	33 (33.7)	40 (41.7)
		≥3 national minimum wage	33 (32.4)	31 (31.6)	34 (35.4)
	On medication, n (%)		91 (89.2)	84 (85.7)	88 (91.7)
	Number of GP visits, median (Q_1_-Q_3_)		5 (2-8)	5 (3-10)	5 (3-8)
**Clinical measures**					
	**Depression severity**				
		BDI-II, mean (SD); median (Min to Max)	22.18 (5.25); 23 (14-28)	22.33 (4.85); 23 (14-28)	22.36 (4.91); 23 (14-28)
	**Perceived health**				
		EuroQol VAS, mean (SD); median (Min to Max)	57.04 (15.77); 57 (20-90)	55.45 (19.23); 50 (10-100)	56.04 (18.34); 60 (0-100)
		Physical Health SF-12, mean (SD); median (Min to Max)	48.87 (11.26); 49.47 (23.52-66.60)	48.52 (11.61); 49.87 (16.76-65.59)	48.60 (11.16); 50.99 (26.12-66.43)
		Mental Health SF-12, mean (SD); median (Min to Max)	28.71 (10.43); 26.68 (10.51-55.69)	28.03 (9.33); 26.16 (5.53-56.02)	28.60 (8.91); 26.83 (14.09-56.09)

### Primary Analyses

There were no clear differences between either CSG or LITG compared with iTAU at Time 1 (Table 2). However, there were differences between iTAU versus CSG, and iTAU versus LITG at Time 2 and at Time 3, with both computerized interventions performing better than usual care. There were no significant differences between CSG and LITG at any time. The adjusted models confirmed these findings (Table 2). Models with missing values replaced through multiple imputations showed small reductions in regression coefficients, but the main differences remained unaltered: Time 2, iTAU vs CSG (B=-2.91; *P*<.001); iTAU vs LITG (B=-3.97; *P*<.001); Time 3, iTAU vs CSG (B=-3.69; *P*<.001); iTAU vs LITG (B=-4.26; *P*<.001). In keeping with the above results, there was a significant group x time interaction (unadjusted: χ^2^_6_=19.23; *P*=.003; adjusted: χ^2^_6_=21.27; *P*=.001; imputed: χ^2^_6_=343.16; *P*<.001). The CACE analysis and the estimated additional intervention effect per sessions attended also showed there was an additional benefit with more sessions attended (Table 4).

**Table 2:** Primary outcome analysis with observed data^a^.

	iTAU (a) mean (SD)	CSG (b) mean (SD)	LITG (c) mean (SD)	*g* (a-b)	*P* (a-b)	B (95% CI) (a-b)	*g* (a-c)	*P* (a-c)	B (95% CI) (a-c)	*g* (b-c)	*P* (b-c)	B (95% CI) (b-c)
BDI-II	n=67	n=57	n=64									
Time 0	21.76 (5.39)	22.59 (4.78)	21.73 (4.83)									
Time 1	17.91 (11.06)	16.59 (10.60)	17.08 (10.24)	0.12	.444	-1.15 (-4.08 to 1.79)	0.08	.634	-0.71 (-3.61 to 2.20)	-0.05	.764	0.44 (-2.45 to 3.34)
Adjusted					.359	-1.35 (-4.23 to 1.54)		.613	-0.74 (-3.60 to 2.12)		.674	0.61 (-2.23 to 3.45)
Time 2	18.12 (12.15)	14.27 (10.00)	13.56 (11.56)	0.34	.007	-4.22 (-7.28 to -1.16)	0.38	.005	-4.34 (-7.36 to -1.33)	0.07	.938	-0.12 (-3.14 to 2.90)
Adjusted					.003	-4.55 (-7.56 to -1.55)		.004	-4.31 (-7.27 to -1.35)		.862	0.26 (-2.70 to 3.22)
Time 3	16.72 (10.97)	11.53 (10.72)	11.39 (10.96)	0.48	.001	-5.10 (-8.20 to -1.99)	0.48	.003	-4.62 (-7.66 to -1.58)	0.01	.758	0.48 (-2.57 to 3.54)
Adjusted					<.001	-5.47 (-8.51 to -2.42)		.002	-4.62 (-7.61 to -1.63)		.574	0.86 (-2.14 to 3.85)

^a^*g*: Hedge’s g as an effect size measure; B: regression coefficients; adjusted: adjusted analysis controlling baseline, sex, and age; a-b: iTAU vs CSG comparison; a-c: iTAU vs LITG comparison; b-c: CSG vs LITG comparison.

### Secondary Analyses

Results with the Mental SF-12 showed a similar pattern of differences as in the primary outcome (Table 3). Analysis with imputed values attenuated coefficients but the main differences were maintained: Time 2, iTAU vs CSG (B=4.79; *P*<.001); iTAU vs LITG (B=4.76; *P*<.001); Time 3, ITAU vs CSG (B=4.63; *P*<.001); iTAU vs LITG (B=5.54; *P*<.001)]. Similarly, group x time interactions were observed (unadjusted: χ^2^_6_=27.05; *P*<.001; adjusted: χ^2^_6_=31.01; *P*<.001; imputed: χ^2^_6_=422.56; *P*<.001), and there were also dose-response effects associated with the number of sessions (Table 4). There were no differences across arms for Physical SF-12 at any time (Table 3). Some differences were found between iTAU vs LITG at Time 2 and Time 3 when using EUROQOL (Table 3), which were confirmed with adjusted and imputed models: imputed Time 1: B=2.49; *P*<.001; Time 2: B=7.26; *P*<.001; Time 3: B=7.66; *P*<.001. At Time 3, there were also EUROQOL differences between iTAU vs CSG in the adjusted model. Using imputed data, these differences were also seen at earlier times (Time 2: B=3.78; *P*<.001; Time 3: B=5.30; *P*<.001). When imputing values, we also found differences between CSG vs LITG at Time 1 (B=2.75; *P*<.001), Time 2 (B=3.48; *P*<.001), and Time 3 (B=2.35; *P*<.001). Group x time effects were not found with unadjusted data (χ^2^_6_=11.10; *P*=.085), but there were interactions with the adjusted (χ^2^_6_=12.88; *P*=.045) and imputed (χ^2^_6_=219.54; *P*<.001) models. Dose-response effects were also found (Table 4).

**Table 3:** Secondary outcome analyses with observed data^a^.

		iTAU (a) mean (SD)	CSG (b) mean (SD)	LITG (c) mean (SD)	*g* (a-b)	*P* (a-b)	B (95% CI) (a-b)	*g* (a-c)	*P* (a-c)	B (95% CI) (a-c)	*g* (b-c)	*P* (b-c)	B (95% CI) (b-c)
**Mental SF-12**		n=64	n=55	n=64									
	Time 0	29.13 (11.18)	28.59 (8.90)	27.95 (8.78)									
	Time 1	35.41 (12.19)	34.72 (12.46)	36.97 (12.57)	0.06	.380	-1.83 (-5.91 to 2.25)	-0.13	.682	0.85 (-3.20 to 4.89)	-0.18	.216	2.63 (-1.54 to 6.80)
	Adjusted					.341	-1.94 (-5.94 to 2.06)		.589	1.10 (-2.87 to 5.06)		.187	3.01 (-1.06 to 7.07)
	Time 2	36.05 (12.38)	42.35 (11.03)	42.22 (13.24)	-0.53	.003	6.49 (2.26 to 10.72)	-0.48	.005	5.97 (1.80 to 10.13)	0.01	.801	-0.56 (-4.89 to 3.78)
	Adjusted					.002	6.67 (2.53 to 10.81)		.002	6.41 (2.33- 10.49)		.863	-0.37 (-4.59 to 3.85)
	Time 3	36.35 (12.12)	43.44 (11.66)	43.65 (13.41)	-0.59	.002	6.78 (2.46 to 11.11)	-0.57	.003	6.53 (2.29 to 10.76)	-0.02	.901	-0.28 (-4.69 to 4.12)
	Adjusted					.001	7.03 (2.80 to 11.26)		.001	7.09 (2.95 to 11.24)		.989	-0.03 (-4.31 to 4.25)
**Physical SF-12**		n=64	n=55	n=64									
	Time 0	48.74 (11.67)	47.83 (12.29)	47.98 (10.87)									
	Time 1	47.91 (10.31)	48.84 (11.89)	49.20 (10.58)	-0.08	.443	1.13 (-1.76 – 4.02)	-0.12	.638	0.69 (-2.17 – 3.54)	-0.03	.760	-0.44 (-3.26 – 2.38)
	Adjusted					.382	1.23 (-1.53 – 4.00)		.542	0.85 (-1.89 – 3.60)		.799	-0.35 (-3.02 – 2.33)
	Time 2	47.56 (10.74)	47.42 (12.18)	46.87 (10.79)	0.01	.820	0.35 (-2.66 to 3.35)	0.06	.906	-0.18 (-3.13 to 2.77)	0.05	.727	-0.52 (-3.46 – 2.41)
	Adjusted					.677	0.61 (-2.26 to 3.47)		.946	0.10 (-2.72 to 2.92)		.727	-0.49 (-3.27 – 2.28)
	Time 3	47.53 (11.78)	47.65 (11.89)	48.05 (9.85)	-0.01	.864	0.27 (-2.81 to 3.34)	-0.05	.655	0.68 (-2.32 to 3.68)	-0.04	.781	0.42 (-2.56 to 3.41)
	Adjusted					.830	0.32 (-2.61 to 3.25)		.620	0.73 (-2.14 to 3.59)		.755	0.45 (-2.37 to 3.27)
**EuroQol VAS**		n=66	n=57	n=65									
	Time 0	57.80 (15.81)	53.56 (20.05)	57.46 (18.23)									
	Time 1	62.12 (18.12)	63.11 (21.61)	65.85 (21.34)	-0.05	.924	0.30 (-5.91 to 6.52)	-0.19	.418	2.55 (-3.61 to 8.70)	-0.13	.495	2.24 (-4.19 to 8.66)
	Adjusted					.816	0.71 (-5.32 to 6.75)		.385	2.66 (-3.33 to 8.64)		.541	1.94 (-4.28 to 8.17)
	Time 2	62.33 (20.83)	65.81 (21.44)	69.83 (20.21)	-0.16	.152	4.73 (-1.73 to 11.19)	-0.36	.016	7.73 (1.38 to 14.09)	-0.19	.381	2.99 (-3.70 to 9.68)
	Adjusted					.088	5.45 (-0.81 to 11.71)		.013	7.84 (1.68 to 14.01)		.478	2.34 (-4.13 to 8.81)
	Time 3	62.59 (20.37)	68.89 (22.79)	72.45 (15.93)	-0.29	.028	7.39 (0.82 to 13.97)	-0.54	.014	8.10 (1.65 to 14.54)	-0.18	.842	0.69 (-6.09 to 7.47)
	Adjusted					.011	8.31 (1.94 to 14.44)		.010	8.19 (1.95 to 14.44)		.970	-0.13 (-6.68 to 6.43)

^a^*g*: Hedge’s g as an effect size measure; B: regression coefficients; adjusted: adjusted analysis controlling baseline, sex, and age; a-b: iTAU vs CSG comparison; a-c: iTAU vs LITG comparison; b-c: CSG vs LITG comparison.

**Table 4:** Dose-response in primary and secondary outcomes.

Variables		Raw^a^		Adjusted^b^		Imputed^c^	
		B	*P*	B	*P*	B	*P*	
**BDI-II**							
	CACE analysis^d^	-6.93 (-12.52 to -1.33)	.016	-7.24 (-12.33 to -2.15)	.006	-8.07 (-9.42 to -6.72)	<.001
	Effect per session	-0.65 (-1.18 to -0.13)	.015	-0.68 (-1.16 to -0.20)	.006	-0.73 (-0.85 to -0.61)	<.001
**Mental SF-12**							
	CACE analysis^d^	9.42 (3.08 to 15.75)	.004	10.27 (4.01 to 16.52)	.001	10.59 (9.05 to 12.12)	<.001
	Effect per session	0.89 (0.29 to 1.48)	.004	0.97 (0.38 to 1.56)	.001	0.95 (0.82 to 1.09)	<.001
**Physical SF-12**								
	CACE analysis^d^	0.01 (-5.71 to 5.74)	.996	0.65 (-3.85 to 5.16)	.776	0.29 (-1.07 to 1.64)	.676
	Effect per session	<1.01 (-0.54 to 0.54)	.996	0.06 (-0.36 to 0.49)	.775	0.03 (-0.10 to 0.15)	.676
**EuroQol VAS**							
	CACE analysis^d^	11.44 (0.76 to 22.12)	.036	12.18 (2.22 to 22.13)	.017	13.12 (10.58 to 15.66)	<.001
	Effect per session	1.08 (0.08 to 2.07)	.034	1.15 (0.22 to 2.07)	.016	1.18(0.96 to 1.41)	<.001

^a^Using raw models.

^b^Adjusting baseline, sex and age.

^c^Using imputed data.

^d^Compliance as attendance at >6 sessions. B: regression coefficients. Controls were considered as receiving 0 sessions.

### Internet-Based Program Usage

At Time 3, 72.4% (n=71) of CSG participants and 75.0% (n=72) of LITG participants had accessed the Internet-based programs (χ^2^_1_=0.35; *P*=.556). In CSG, the median number of modules completed was 4 (interquartile range: 0-10), with 41.8% attending >6 modules; 30 participants in this group completed sessions. In LITG, the median number of modules completed was 6 (interquartile range: 0-10), with 50.0% attending >6 modules and 40 participants completing 10 modules. There was no significant difference between CSG and LTIG in terms of modules completed (Z=-1.20; *P*=.228). A total of 17 email contacts were made with 13 participants of the LITG program, which represented 11.9% of the patients in this group. Support requests were monitored, and a content analysis of them showed that the topics were related to (1) requesting information on depressive symptoms, (2) counseling with regard to events and difficulties in life, and (3) additional support to follow the program recommendations. As per protocol, CSG received no email contact from therapists.

### Other Treatments Received

Most of the participants received drug treatment (Table 1). There were no major differences in the use of medication (as a dichotomous criterion of drug intake) across groups (iTAU: 80.6%; CSG: 68.3%; LITG: 67.6%; χ^2^_2_=3.67; *P*=.160). The same pattern was found for the use of mental health services, psychiatrist and/or psychologist (iTAU: 29.9%; CSG: 19.7%; LITG: 18.8%; χ^2^_2_=3.09; *P*=.214), and number of visits to the GP [iTAU: median=2 (Q_1_=0; Q_3_=4); CSG: median=1 (Q_1_=0; Q_3_=3); LITG: median=1 (Q_1_=0; Q_3_=4); χ^2^_2_=0.22; *P*=.643].

## Discussion

### Principal Findings

As far as we are aware, this is the first RCT on the effectiveness of an Internet-based intervention for the treatment of depression in PC health services in Spain. Other Web-based interventions for treating depressive symptoms in the Spanish language have been developed in Mexico [37] and Chile [38], but the efficacy of these interventions in reducing depressive symptoms has not yet been evaluated in an RCT. Therefore, this trial is novel in that it allowed the testing of a new Spanish program (Smiling is Fun), which adapted some of the techniques used in other available programs [24,39]. This trial compared two Internet-based interventions, with and without psychotherapeutic support, with usual care. We observed differences in the medium and long term in favor of psychotherapy, but not in the short term. This was somewhat consistent with previous studies on depression when comparing face-to-face psychotherapy (alone or plus usual care), with usual care based on medication treatments [40]. We found a larger effect size than in previous works, particularly in the group without additional support, and we also found that the offer of support did not yield additional benefits in terms of better adherence or outcomes, contrary to other previously published studies [41,42].

The way patients were recruited in PC settings, with face-to-face contacts in a confidential context with the involvement of the GP, might explain why our results were better than those of other trials [14]. Studies in which patients suffering from depression were referred by their GP for treatment had generally shown higher effect sizes, with values similar to our study [5]. This aspect of the trial may have also contributed to the low number of email contacts requesting assistance to the psychotherapists, and therefore, to the similarity in the results for the supported and the unsupported groups. This same result has also been found in other works [15], although effect sizes in Internet-based interventions with therapist support typically seem larger than those without therapist support [14]. In our study, the type of support received by email on request was less intense than in other efficacy trials, which additionally include a group with some kind of weekly contact [14,15]. Patients in our study did not make much use of the support offered. We do not know the real reasons for this, but the lack of initial face-to-face contact with the psychotherapist might have hampered the establishment of an adequate alliance—something that might merit interest for further studies [19].

The possibility of receiving attention and care from a GP in a PC setting could also help minimize the stigma associated with referral to mental health services [11] and could improve adherence to the program. In fact, our procedure was as close as possible to the usual practice, and thus, it might increase the likelihood that the program might be used in the PC health services in Spain, where most depressed patients are treated [43]. Nevertheless, GPs may experience difficulties in recruiting patients because of their overloaded schedule, as has been described in other countries [44].

### Limitations

This study encountered several limitations. First, attrition at follow-up was significant, but replacing missing values through imputations confirmed the main findings. As shown in other similar trials of computerized interventions, attrition rates are often large [12,32]. If anything, our retention rate was probably as good, if not better, than that achieved in other similar trials. Second, the potential effect of GPs or psychotherapist was not taken into account and may have been a source of variability. Finally, this trial was not powered to detect small differences between the two computerized interventions. This part of the study needs to be tested properly in a separate trial also controlling for the possible effects of psychotherapists and GPs.

### Conclusions

A Spanish-language Internet-based intervention for the treatment of depression (Smiling is Fun) added to usual care proved to be more effective than treatment as usual alone at follow-up assessments. Pending cost-effectiveness analysis, these results suggest that it might be worth investing in this program for PC clinics in Spain, and possibly in other Spanish-speaking settings. The kind of low-intensity support offered in the program did not show additional improvement on the effectiveness of the computerized intervention. It remains to be seen whether or not any other forms of online/telephone support might yield further gains.

## Appendix

Multimedia Appendix 1 CONSORT-EHEALTH checklist V1.6.1 [33].

## Abbreviations

BDI-II: Beck Depression Inventory-II
CACE: Complier Average Causal Effect
CSG: completely self-guided Internet-based program
DSM-IV: Diagnostic and Statistical Manual, version IV
GP: general practitioner
iTAU: improved treatment as usual care
LITG: low-intensity therapist-guided Internet-based program
PC: primary care
RCT: randomized controlled trial
SF-12: Short Form Health Survey
